# Plant regeneration from cell suspension culture in *Saccharum officinarum* L. and ascertaining of genetic fidelity through RAPD and ISSR markers

**DOI:** 10.1007/s13205-016-0579-3

**Published:** 2017-04-08

**Authors:** Avinash S. Thorat, Nishant A. Sonone, Vrushali V. Choudhari, Rachayya M. Devarumath, K. Harinath Babu

**Affiliations:** 1 Molecular Biology and Genetic Engineering Section, Vasantdada Sugar Institute, Manjari (Bk), Pune, Maharashtra India; 2 Department of Botany, Shivaji University, Kolhapur, Maharashtra India

**Keywords:** Sugarcane, Callus, Cell suspension culture, RAPD, ISSR, Somaclonal variation, Genetic fidelity

## Abstract

The aim of this study was to produce sugarcane plantlets from cell suspension culture and study its genetic fidelity using molecular markers. The study was carried out using sugarcane varieties Co 86032 and Q117. Callus cultures of both the varieties were optimized using six different callus induction media. After screening the growth response of callus on six different callus induction media, it was observed that medium no. VI supplemented with 500 mg l^−1^ of each PVP, Casein hydrolysate and MES buffer showed high amounts of callus in Co 86032 (79.66 ± 0.44%) and Q117 (82.83 ± 1.69%). Addition of PEG 8000 at 2.5% to this medium had a profound impact on inducing somatic embryogenesis in Co 86032 (54.66 ± 1.76%) and Q117 (66.66 ± 2.60%) as compare to control (24.33 ± 1.76%) and (27.33 ± 2.73%), respectively. Cell suspension cultures were established by culturing embryogenic calli in liquid medium showed well established suspension cultures with fever cell aggregates. There was negligible cell division during initial 2 days of incubation and cell count increased rapidly between 2 and 8 days. Further incubation beyond 8 days resulted in a decrease in cell viability. Enhanced callus proliferation in Q117 while enhanced shoot regeneration in Co 86032 was observed from cell suspension culture. The clonal fidelity of in vitro regenerated plants was assessed by using RAPD and ISSR markers. Analysis of the ten RAPD markers indicated that 90.48 and 86.95% true-to-type regenerated plantlets in Co 86032 and Q117, respectively. However, in the ISSR markers, Co 86032 did not show any polymorphism and in the Q117, 92.18% true-to-type plantlets were found. These results confirmed that somaclonal variation occurs during the process of indirect organogenesis and RAPD and ISSR marker based molecular analysis is a suitable method for an early detection of variation in sugarcane.

**Electronic supplementary material:**

The online version of this article (doi:10.1007/s13205-016-0579-3) contains supplementary material, which is available to authorized users.

## Introduction

Sugarcane (*Saccharum officinarum* L.), is an important cash crop which contributes to around 70–80% of sugar production globally. It is cultivated in the tropical and sub-tropical regions and ranks amongst the top ten cultivated crops in the world (Suprasanna et al. 2011). In India, sugarcane has secured a distinct position after cotton as an agro-industrial crop, due to it being a prominent source of vital product (sugar) as well as by-products (baggasse, molasses, pressmud, etc.) which plays a major role in the economic progress of small and large scale industrial sectors. Sugarcane has been recognized as the most competent crop which converts solar energy into harvestable chemical energy in the form of sucrose and biomass (Joyce et al. 2010).

Currently, the development of somatic embryogenesis through callus and suspension culture has great potential for propagation at a rapid rate (Dewanti et al. 2016). It was reported that all the regenerated plants from the tissue culture are not true-to-type like the parent. Phenotypic variation is commonly observed amongst regenerated plants which are associated with genetic changes of an organism. These changes could result from mutations, epigenetic changes or a combination of both mechanisms. Somaclonal variation is the genetic variability, which has occurred during tissue culture or variants derived from any form of cell or tissue cultures (Larkin and Scowcroft 1981). The occurrence of somaclonal variation arising through cryptic gene defects can seriously limit the application of micropropagation for clonal multiplication (Rani and Raina 2000). These variations in genomic DNA of regenerants restrict the utility of plant micropropagation techniques for large-scale multiplication. The inability for quick identification of these polymorphisms at cytological, biochemical, phenotypic and molecular levels in micropropagated sugarcane poses a challenge. The rapid detection is beneficial to check the homogeneity between mother and in vitro grown plantlets (Devarumath et al. 2002). The PCR associated DNA molecular markers based analysis system such as Random amplified polymorphic DNA (RAPD), Amplified fragment length polymorphism (AFLP), Simple sequence Repeat (SSR), Inter-sequence simple repeats (ISSR) and microsatellite DNA/SSRs possess several benefits over the traditional methods that have been used for detection of polymorphism and genotyping in plant systems (Lal et al. 2008; Guasmi et al. 2012; Rajpal et al. 2014). The RAPD and ISSR analysis has been widely used to determine polymorphism in genomic DNA in sugarcane and several other plant systems due to numerous advantages like being a relatively fast, simple, cost-effective technique which requires a small quantity of DNA sample with no preliminary sequence information for primer design (Suprasanna et al. 2006; Devarumath et al. 2007; Lal et al. 2008; Rizvi et al. 2012; Dangi et al. 2014; Kshirsagar et al. 2015).

The present work was performed with an objective to establish sugarcane (Co 86032 and Q117) suspension culture and subsequent regeneration of plantlets and study their genetic variation occurred during the process of regeneration using molecular markers (RAPD and ISSR).

## Materials and methods

### Plant materials and explants preparation

The sugarcane varieties Co 86032 and Q117 grown in experimental farms of Vasantdada Sugar Institute, Manjari (Bk.), Pune, Maharashtra, India were used in this investigation. The sugarcane tops were harvested from 4 to 6 months old field grown sugarcane varieties Co 86032 and Q117. The harvested shoot tops (25–30 cm) were cleaned with a surfactant Teepol™ (0.1%) for 4–5 min followed by a rinse under tap water for 10–12 min. The outer mature leaves of shoot tops were removed and discarded. The shoot tops were then surface sterilized using Bavistin™ (0.1%) and streptomycin (0.1%) for 15 min followed by HgCl_2_ (0.1%) for 30 min and then rinsed with sterile distilled water for 2–3 times.

### Initiation of callus

The immature leaf whorls from sterile cane tops were sliced (about 1–2 mm thickness) and inoculated on six different callus induction medium (Table 1) and incubated in dark at 28 ± 2 °C for 2 weeks. The medium showing higher proliferation of callus from Co 86032 and Q117 was selected for somatic embryogenesis and subculture was performed after 2 weeks of inoculation. The callus induction response (%) for each medium was recorded as:

$${\text{Callus}}\;{\text{induction}}\;(\% ) = \frac{{{\text{Number}}\;{\text{of}}\;{\text{calli}}\;{\text{induced}}}}{{{\text{Number}}\;{\text{of}}\;{\text{leaf}}\;{\text{whorls}}\;{\text{inoculated}}}} \times 100$$

**Table 1:** Media combination tested for callus induction

Component (l^−1^)	I	II	III	IV	V	VI
NH_4_NO_3_(g)	1.65	1.65	1.65	1.65	1.65	1.65
CaCl_2_·2H_2_O (mg)	440	440	440	440	440	440
MgSO_4_·7H_2_O (mg)	370	370	370	370	370	370
KH_2_PO_4_ (mg)	170	170	170	170	170	170
KNO_3_ (g)	1.9	1.9	1.9	1.9	1.9	1.9
Na_2_EDTA (mg)	37	37	37	37	37	37
FeSO_4_ (mg)	28	28	28	28	28	28
H_3_BO_3_ (mg)	6	6	6	6	6	6
CoCl_2_·6H_2_O (µg)	25	25	25	25	25	25
CuSO_4_·5H_2_O (µg)	25	25	25	25	25	25
MnSO_4_·7H_2_O (mg)	22	22	22	22	22	22
KI (mg)	1	1	1	1	1	1
Na_2_MoO_4_·2H_2_O (µg)	250	250	250	250	250	250
ZnSO_4_·7H_2_O (mg)	9	9	9	9	9	9
2,4-D (mg)	3	3	3	3	3	3
Sucrose (g)	–	40	30	30	30	20
Maltose (g)	30	–	–	–	–	–
Coconut water (ml)	–	–	100	100	100	100
l-Glutamine (mg)	–	100	–	30	–	–
Kinetine (mg)	0.5	1	2	–	–	–
NAA (mg)	–	0.5	0.5	–	–	–
PVP (mg)	100	100	100	100	100	500
Casein hydrolysate (mg)	–	–	–	50	–	500
MES buffer (mg)	–	–	–	–	–	500
Thiamine HCl (mg)	–	–	–	–	–	1
Inositol (mg)	–	–	–	–	–	20
Proline (mg)	500	–	–	50	–	500

### Effect of PEG on somatic embryogenesis

The proliferated calli of Co 86032 and Q117 were transferred to medium with varying concentration of polyethylene glycol 8000 (PEG) (0, 2.5, 5.0, 7.0 and 10.0%) and incubated in dark at 28 ± 2 °C for 2 weeks. The effect of PEG on somatic embryogenesis was examined by measuring the number of somatic embryos formed after 2 weeks. The somatic embryogenesis (%) for each combination was recorded as:

$${\text{Somatic}}\;{\text{embryogenesis}}\;(\% ) = \frac{{{\text{Number}}\;{\text{of}}\;{\text{somatic}}\;{\text{embryos}}\;{\text{generated}}}}{{{\text{Number}}\;{\text{of}}\;{\text{calli}}\;{\text{initiated}}}} \times 100$$

### Development of cell suspension culture

Cell suspension culture was developed from 8 weeks old embryonic callus (2–2.5 g fresh weight) in liquid MS medium (Murashige and Skoog 1962) (20 ml) supplemented with coconut water (5% v/v), 2,4-D (3 mg l^−1^) and casein hydrolysate (500 mg l^−1^) and pH was adjusted to 5.8 ± 0.1 before autoclave. The culture bottles were continually agitated at 100 rpm and incubation in dark at 28 ± 2 °C. After 8–10 days, suspension culture was transferred to fresh medium.

### Growth kinetic studies of cell suspension culture

In suspension culture growth of the cells was calculated by measuring cell density using spectrophotometer (OD_600_) (Mohler et al. 1996) and by haemocytometer (Aberkane et al. 2002). Cell density was measured after every 24 h interval for nine subsequent days from initial culture. The total number of cells/ml was calculated according to the method explained by Dodds and Roberts (1986) and cell viability was determined by using trypan blue exclusion test (Katsares et al. 2009) using the following formulas

$${\text{Total}}\;{\text{number}}\;{\text{of}}\;{\text{cells}}/{\text{ml}} = \frac{{{\text{Total}}\;{\text{cells}}\;{\text{counted}}}}{{{\text{Total}}\;{\text{squares}}\;{\text{counted}}}}\; \times {\text{Dilution}}\;{\text{factor}}\; \times \;10^{4}$$

$${\text{Number}}\;{\text{of}}\;{\text{viable}}\;{\text{cells}}/{\text{ml}} = \frac{{{\text{Total}}\;{\text{unstained}}\;{\text{cells}}\;{\text{counted}}}}{{{\text{Total}}\;{\text{squares}}\;{\text{counted}}}}\; \times {\text{Dilution}}\;{\text{factor}}\; \times \;10^{4}$$

### Plant regeneration from suspension culture

Cells were harvested from suspension for callus induction by filtering the suspension using whatmann filter paper no. 1. The filter paper containing harvested cells was carefully inoculated in petri plates containing callus induction medium supplemented with PEG 8000 (2.5%) and incubated at 26 ± 2 °C under dark for the development of embryonic callus. The regeneration capacity of callus was determined by inoculating cell suspension and cell aggregates onto shoot regeneration medium [MS basal medium fortified with benzylaminopurine (1 mg l^−1^), kinetin (0.5 mg l^−1^)] and incubated at 26 ± 2 °C and 16/8 h (light/dark) photoperiod regimes.

### Root induction and acclimatization

Root induction was accomplished by incubating elongated shoots (4–5 cm) from callus on to MS basal medium augmented with naphthalene acetic acid (0.5 mg l^−1^) and incubated at 26 ± 2 °C with the 16 h photoperiod. Plantlets with well-developed roots were washed with water and transferred to plastic cups containing a mixture of soil, sand and coco peat (3:1:1) and incubated as above mentioned conditions. After a week of incubation, these plantlets were subsequently potted in soil and maintained under greenhouse conditions.

### DNA extraction

Total genomic DNA of young leaves of the mother plant and in vitro raised plants were isolated using Aljanabi et al. (1999) method. The quality of genomic DNA was resolute on 0.8% agarose gel electrophoresis, while quantity by UV–Vis spectrophotometry (UV-1700, Shimadzu Corporation, Japan). The final concentration of extracted genomic DNA was made to 50 ng µl^−1^ and stored at −20 °C till further use.

### Genetic stability analysis using molecular markers (RAPD and ISSR)

Two sets of primers including arbitrary (RAPD) and semi arbitrary (ISSR) were used for analysis of genomic DNA including mother plant and in vitro raised plants. Ten primers each from RAPD (OPH series, Operon Technologies, INC. California, USA) (Table 2) and ISSR (UBC series, University of British Columbia, Vancouver, Canada) (Table 3) were selected on the basis of preliminary screening. PCR analysis was done by the method explained by Williams et al. (1990) with slight modifications. For RAPD analysis, DNA amplification was carried out with total reaction mixture volume of 20 µl consisting 50 ng template DNA (1 µl), 10× PCR buffer with MgCl_2_ (2 µl), 250 µM dNTPs (2 µl), 0.25 µM primer (2 µl), 1 U Taq DNA polymerase (0.2 µl) and sterile nucleus free distilled water (12.8 µl). PCR was performed on Thermal cycler (Applied Biosystem) at initial temperature of 94 °C (5 min, 1 cycle), followed by 40 cycles of 1 min at 94 °C, 1 min at 37 °C, 2 min at 72 °C and final extension cycle of 10 min at 72 °C. During ISSR analysis, the reaction mixture was made similar to RAPD. However, PCR was carried out using initial denaturation at 95 °C for 5 min followed by 40 cycles 1 min at 94 °C, 1 min at 52–54 °C (depending on the primer), 2 min at 72 °C and final extension cycle of 10 min at 72 °C. The PCR products were stored at 4 °C until further analysis carried out. The PCR products were resolved on 1.5% (w/v) agarose gel with 1× TBE buffer, stained with ethidium bromide (EtBr) and documented under UV light. The fragment size was estimated using 1 kb DNA ladder.

**Table 2:** Molecular polymorphism analyzed by 10 RAPD markers in sugarcane variety Co 86032 and Q117

Sr. No	Primer code	Primer sequence (5′–3′)	Co 86032			Q117			Size range (bp)
			*n*SB	*n*MB	*n*PB	*n*SB	*n*MB	*n*PB	
1	OPH-01	GGTCGGAGAA	5	5	0	5	4	1	250–3000
2	OPH-02	TCGGACGTGA	5	5	0	6	6	0	250–3000
3	OPH-04	GGAAGTCGCC	5	5	0	6	5	1	250–2000
4	OPH-06	ACGCATCGCA	5	5	0	8	7	1	250–3000
5	OPH-09	TGTAGCTGGG	4	2	2	3	3	0	250–1000
6	OPH-11	CTTCCGCAGT	6	6	0	6	6	0	250–1000
7	OPH-13	GACGCCACAC	5	5	0	5	4	1	250–2000
8	OPH-16	TCTCAGCTGG	2	2	0	3	3	0	500–2000
9	OPH-17	CACTCTCCTC	2	2	0	2	2	0	500–1000
10	OPH-18	GAATCGGCCA	2	1	1	2	0	2	250–500
**Total**			**42**	**38**	**4**	**46**	**40**	**6**	–
**Percentage (%)**				**90.48**	**9.52**	–	**86.95**	**13.05**	–

**Table 3:** Molecular polymorphism analyzed by 10 ISSR markers in sugarcane variety Co 86032 and Q117

Sr. No.	Primer code	Primer sequence (5′–3′)	Co 86032			Q117			Size range (bp)
			*n*SB	*n*MB	*n*PB	*n*SB	*n*MB	*n*PB	
1	UBC-811	(GA)8C	5	5	0	5	4	1	50–1500
2	UBC-828	(TG)8A	4	4	0	6	6	0	50–1500
3	UBC-835	(AG)8YC	7	7	0	5	3	2	50–3000
4	UBC-836	(AG)8YA	7	7	0	7	7	0	50–1000
5	UBC-844	(CT)8RC	6	6	0	8	8	0	50–1500
6	UBC-849	(GT)8YA	6	6	0	6	5	1	50–1000
7	UBC-855	(AC)8YT	5	5	0	6	6	0	50–1500
8	UBC-857	(AC)8YG	8	8	0	9	8	1	50–1500
9	UBC-864	(ATG)6	5	5	0	7	7	0	50–1500
10	UBC-868	(GAA)6	9	9	0	5	5	0	50–2000
**Total**			**62**	**62**	**0**	**64**	**59**	**5**	–
**Percentage (%)**			–	**100**	**0**	–	**92.18**	**7.81**	–

*nSB* total number of scorable bands, *nMB* number of monomorphic bands, *nPB* number of polymorphic bands

### Statistical analysis

All experiments were performed in duplicate, with the three independent replicates, as well as different DNA extraction of the same bulk sample to maintain the consistency of results. The data was analyzed using Microsoft Excel 2010 and SPSS for Windows (version 16). One way ANOVA was applied to test mean differences of all treatments while a statistical significant difference between mean values was established at *p* ≤ 0.05 while Duncan’s New Multiple Range Test was used. The results were expressed as mean ± SE. For RAPD and ISSR analysis, a band with the same mobility were counted as an identical band while each amplified product was scored as present (1) or absent (0), bands of low intensity, which was difficult to be distinguished as present or absent were not considered.

## Results and discussion

### Impact of culture medium on induction of callus

**Fig. 1:**
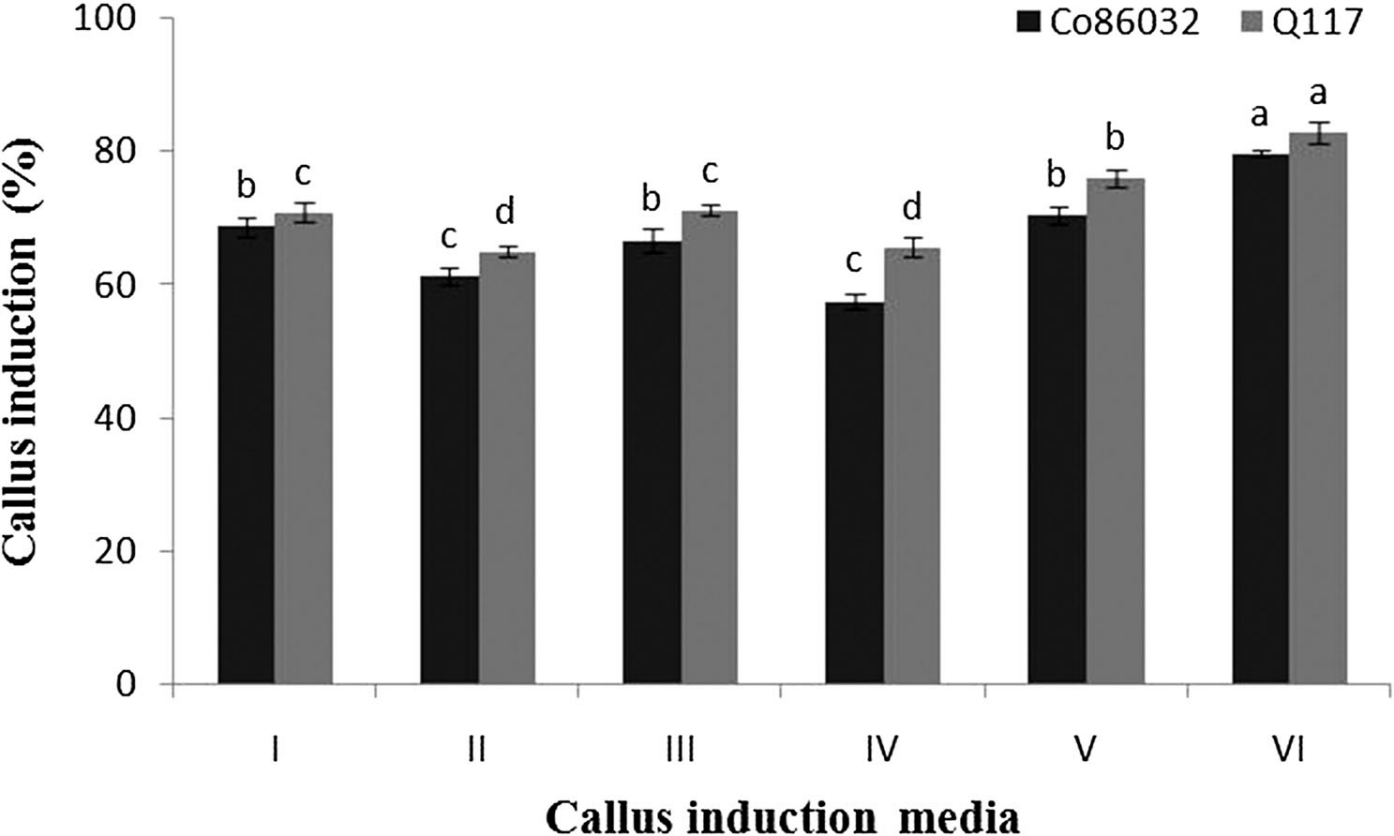
Callus induction response of Co 86032 and Q117 to different media

Inoculated leaf whorl disks from both the varieties (Co 86032 and Q117) shows variation in quality and rate of callus generated in different media. During incubation browning of some disks due to secretion of diffusible polyphenolic compounds was noticed. Significantly superior callus induction of Co 86032 and Q117 variety was noticed in callus induction medium number VI (79.66 ± 0.44 and 82.83 ± 1.69%), while least callus induction was observed in medium number IV (57.50 ± 1.04 and 65.66 ± 1.45%) (Fig. 1). This might be due to the fortification of an appropriate concentration of thiamine, casein hydrolysate, MES buffer, proline, PVP and inositol along with other nutrients in callus induction medium number VI (Table 1). These supplemented components in specified concentration and in combination with each other, while other media components are known to augment the callus induction. The excess amount of PVP (500 mg l^−1^) restricted the phenolics secretion by leaf whorl callus into the culture medium. This is due to the chelating ability of PVP which binds ions responsible for activation of polyphenol oxidative enzymes and these results are in agreement with previous reports of sorghum tissue culture by Liu et al. (2015). Thiamine, which serves as a cofactor in numerous metabolic pathways is essential for the growth of all cells in tissue (Goyer 2010). Inositol stimulates the cell growth and has a vital role in cell division and hence added in a small quantity of the growth medium (Saad and Elshahed 2012). Casein hydrolysate used in the culture medium is a mixture of amino acids, which are the ambient source of organic nitrogen. The medium supplemented with casein hydrolysate has growth promoting activity on different genotypes of rice (Visarada et al. 2002) while the stimulatory effect on embryo initiation and callus regeneration in sugarcane Nasir et al. (2011). MES is a buffering agent added to stabilize the pH of growth medium (de Klerk et al. 2008) while proline as a compatible solute which plays a critical osmoprotective role in numerous physiological responses, enabling plants to bear the undesirable effects of abiotic stress (Nounjan et al. 2012). The medium fortified with MES and proline had a significant impact on cell proliferation during growth of rice suspension culture (Kermanee 2004).

### Determination of callus type

**Fig. 2:**
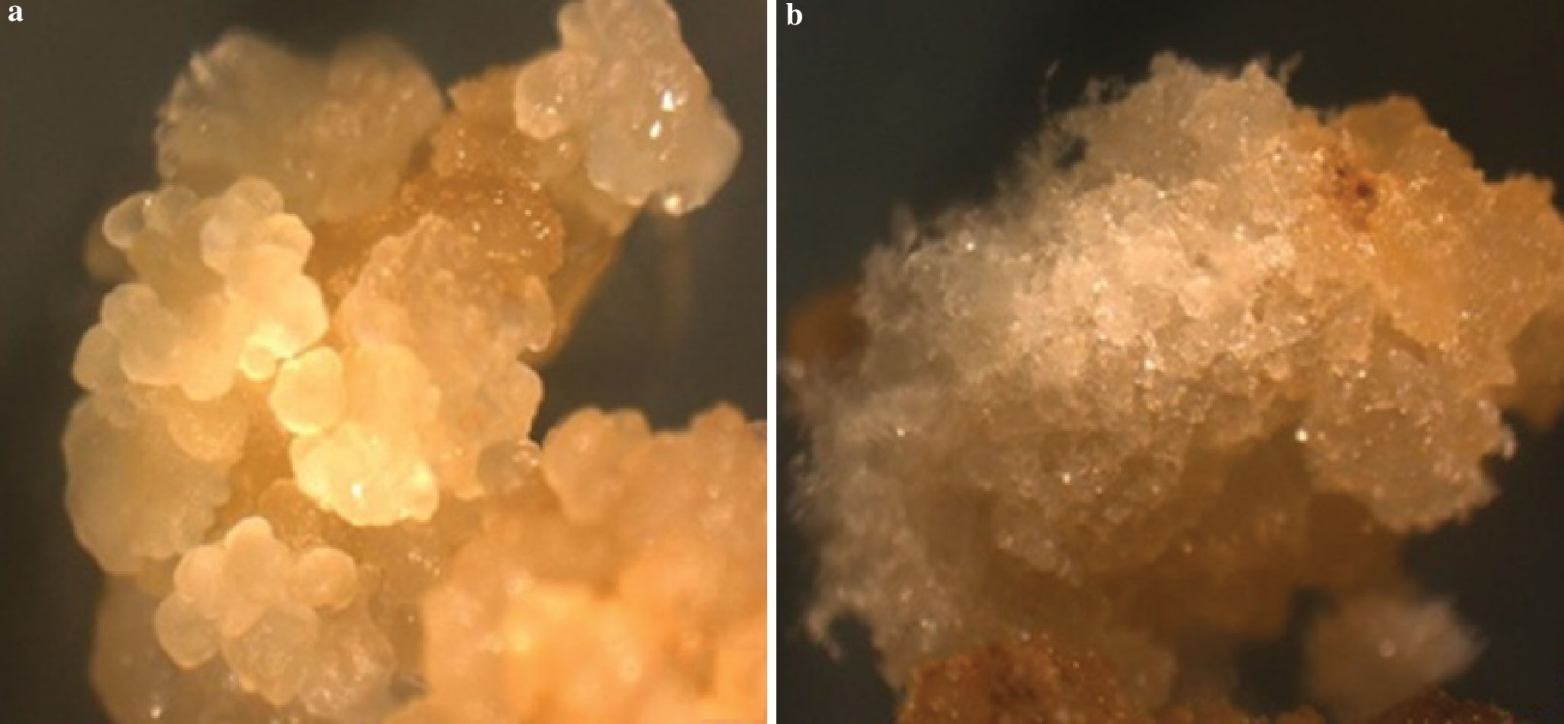
Type of callus: **a** compact, friable, creamish and embryogenic and **b** loose, filamentous, whitish, watery and non-embryogenic

The calli developed in the induction medium was phenotypically heterogeneous and hence differentiated visually based on morphology into two types i.e. embryogenic (compact, friable, creamy and embryogenic) and, non-embryogenic (loose, filamentous, whitish, watery and non-embryogenic) (Fig. 2). The embryogenic callus facilitated the development of somatic embryos. The generation of embryogenic callus along with a rate of callus induction was more prominent in Q117 than Co 86032. In some plates embryogenic and non-embryogenic type of calli developed from the same explant and somatic embryos formed on cut edges of leaves. In-vitro embryogenesis was triggered by physiological and genetic factors. These findings have supported the results of Basnayake et al. (2011) and Silveira et al. (2013). Additionally, plant growth hormones (e.g. Abscisic acid, auxins like 2,4-D, etc.) along with explants source, carbohydrates, and nitrogen source plays a crucial role for initiation or inhibition in embryogenic cells and tissues (Dewanti et al. 2016).

### Effect of PEG on somatic embryogenesis

**Fig. 3:**
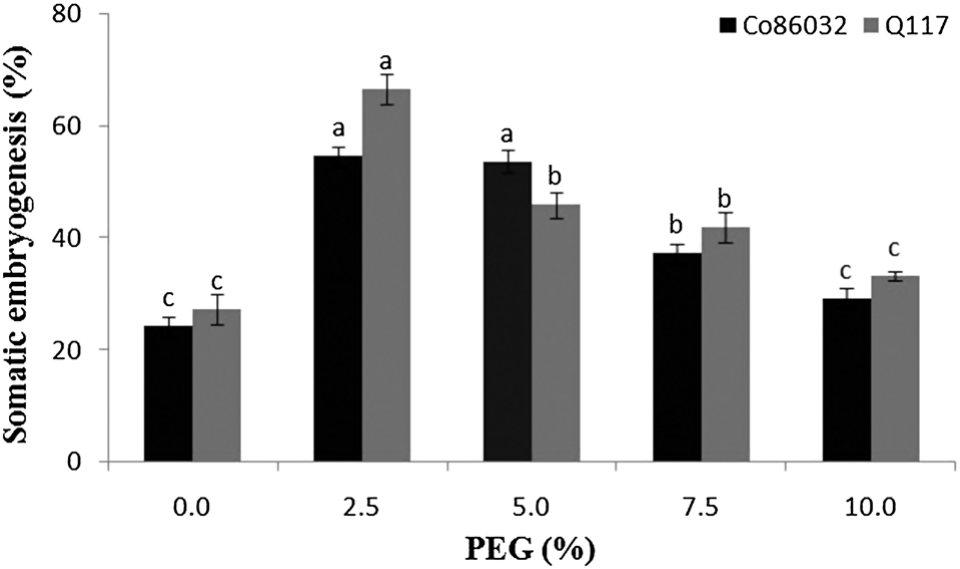
Effect of PEG on somatic embryogenesis in Co 86032 and Q117

The effect of PEG 8000 on development and growth of somatic embryos was determined by incorporating PEG 8000 at a different concentration in the callus induction medium. PEG 8000 had a considerable impact on the development of somatic embryos in Co 86032 (54.66 ± 1.76%), Q117 (66.66 ± 2.60%) at 2.5% PEG. However, in the case of control (medium without PEG 8000) only 24.33 ± 1.76 and 27.33 ± 2.73% of embryos were observed respectively (Fig. 3). The sugarcane variety Co 86032 performed an almost equal percentage of somatic embryogenesis at 2.5 and 5% concentrations of PEG 8000 in the medium. At lower concentration, PEG along with sucrose controls cellular necrosis enhancing somatic embryogenesis (Rizvi et al. 2012). At the above concentration of 5%, the growth gradually reduced because of stress conditions developed by PEG 8000 due to binding of nutrients and free water required for cellular metabolism. Moreover, PEG 8000 at higher concentrations developed the high osmotic potential in the culture medium because of accumulation of minerals around it.

### Initiation and maintenance of cell suspension culture

The cell suspension culture was initiated by inoculating calli in liquid MS basal medium fortified with coconut water (500 ml l^−1^), 2,4-D (3 mg l^−1^) and casein hydrolysate (500 mg l^−1^) and cultures were maintained on continuous shaking at 100 rpm. During incubation shaking serves both, to aerate the culture and to disperse the eel in medium (Shah and Seth 2010). The initiation of cell suspension culture requires comparatively a good quantity of well-differentiated callus mass to serve as inoculums. Due to continuous agitation, the actively dividing and newly formed cells gradually free themselves from the inoculated fragile callus (Parekh et al. 2008). The well-established suspension cultures with no cellular clumps were passage into fresh medium and incubated under constant agitation for maintaining viable cells in a free form.

### Determination of growth in suspension culture

**Fig. 4:**
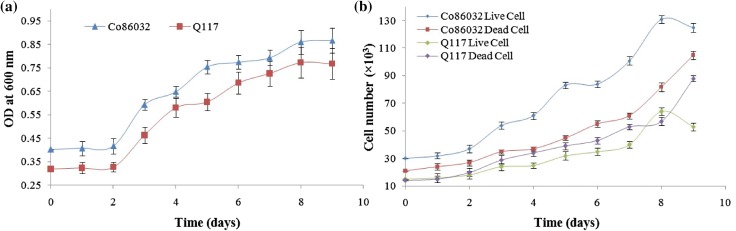
Cell suspension culture growth of Co 86032 and Q117 by spectrophotometer (OD_600_) (**a**) and by haemocytometer (**b**)

The growth of sugarcane varieties Co 86032 and Q117 cell density in suspension culture was measured by the spectrophotometric method at OD_600_ and cell size by haemocytometer, while cell viability was estimated using tryphan blue dye exclusion method. The growth curve plotted using OD_600_ and cell count is shown in Fig. 4a, b and it was noticed that cell dividing rate is faster in Co 86032 as compared to Q117. There was negligible cell division (as OD_600_ and cell count was constant) during first 2 days of incubation due to the acclimatization of cells to the media components and growth conditions. This was followed by vigorous cell division (as OD_600_ and cell count increased rapidly) between 2 and 8 days. Further incubation beyond 8 days resulted in the decrease in cell viability due to depletion of nutrients and it comes to stationary phase. (Ho and Vasil 1983) also reported the similar results and observed a maximum number of cells on the 8th day of culture. Hence longstanding medium needs to be replaced for maintaining a high live cell count (Shah and Seth 2010).

### Determination of cell type and size

The cell suspension culture was composed of two basic types of cells, i.e. embryogenic: spherical cells with prominent and actively dividing nucleus, rich in cytoplasm and starch granules and non-embryogenic: cylindrical cells with large vacuoles (Supporting Figure 1). The cell size measured with ocular micrometry indicated it to be in between 5 and 10 µm in the case of embryogenic cells and 10–45 µm in the case of non-embryogenic cells in both Co 86032 and Q117 tissues. The embryogenic and non-embryogenic cells were developed in the same cultures, however, non-embryogenic cells were comparatively higher in number than embryogenic cells. The number of embryogenic cells in Q117 was observed higher than Co 86032. The similar evidence of embryogenic and non-embryogenic cells was reported in sugarcane cell suspension culture in clone 68–1067 by Ho and Vasil (1983) and Falco et al. (1996).

### Plant regeneration from cell suspension culture

**Fig. 5:**
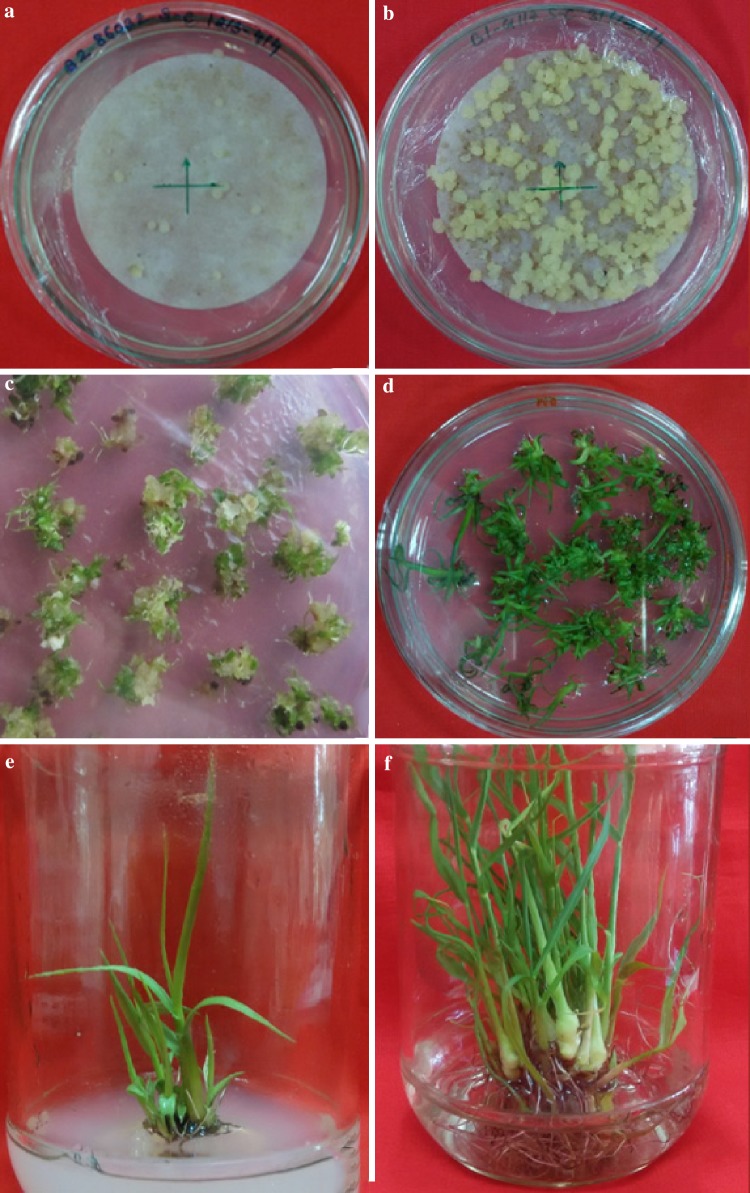
Different stages of plant regeneration through cell suspension culture in *S. officinarum* (Co 86032 and Q117). **a** suspension culture on callus induction medium [MS + 3 mg l^−1^ 2,4-D + 20 g/l sucrose + 100 ml l^−1^ coconut water + 500 mg l^−1^ PVP + 500 mg l^−1^ casein hydrolysate + 500 mg l^−1^ MES buffer + 1 mg l^−1^ thiamine HCL + 20 mg l^−1^ inositol + 500 mg l^−1^ proline + 2.5% PEG (8000)]. **b** Callus proliferation after 15 days of suspension culture, **c** shoot regeneration [MS + 1 mg l^−1^ BAP, 0.5 mg l^−1^ kinetin], **d** regenerated plantlets, **e** plantlets on root induction medium [MS + 5 mg l^−1^ NAA], **f** fully developed plantlets on shoot elongation medium

**Fig. 6:**
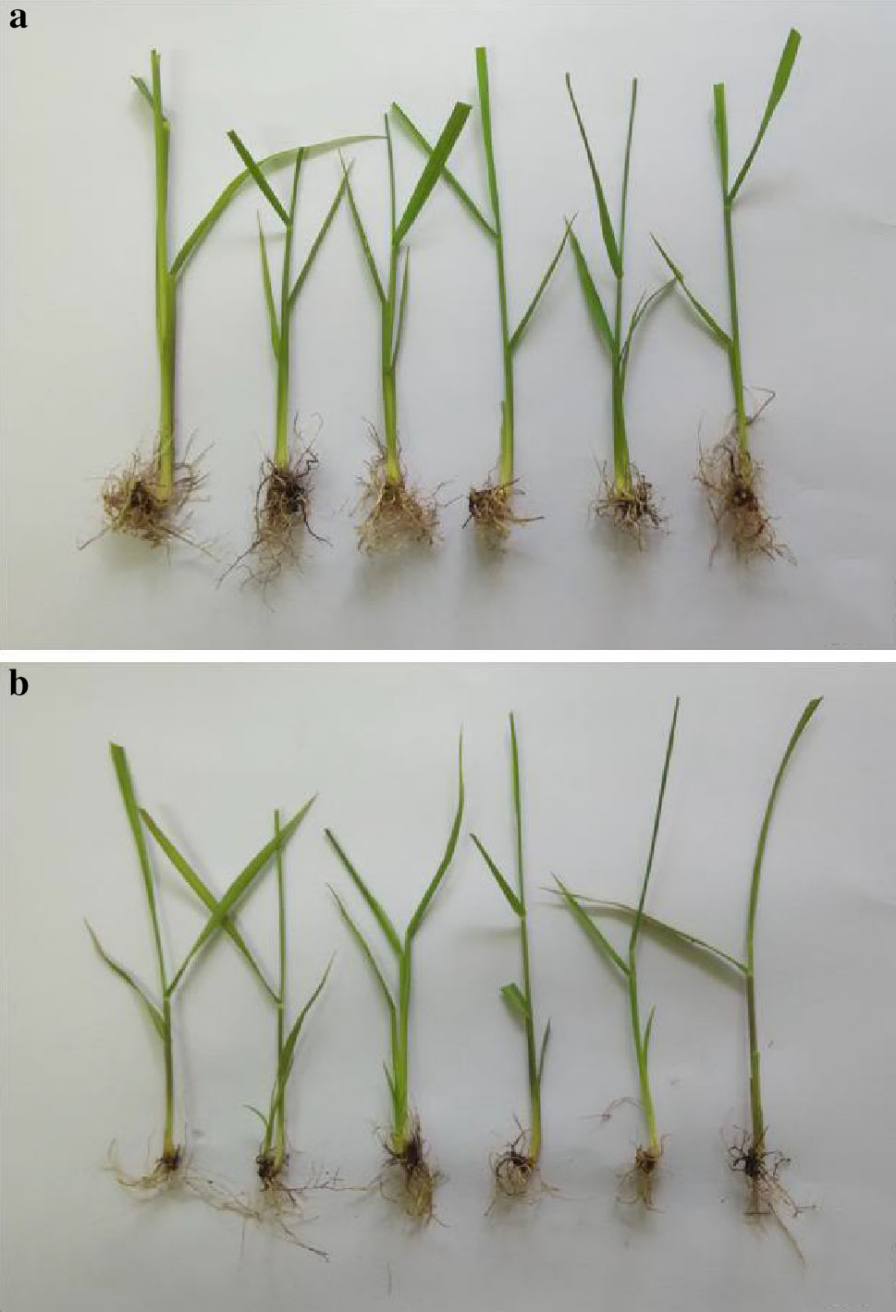
Rooting profile of regenerated plants of sugarcane. **a** Co86032 and **b** Q117

Callus induction of Co 86032 and Q117 from suspension culture achieved by inoculation of cells harvested from suspension culture into the callus induction medium VI supplemented with PEG 8000 (2.5%) is shown in Fig. 5a, b. It was observed that the number of Q117 calli induced and proliferated were comparatively more, prominent and actively dividing in contrast with Co 86032 (Fig. 4a, b). The possible reason for this could be the presence of potentially dividing cells with prominent nuclei (embryogenic) in Q117 callus as compared to Co 86032. The callus induced from the harvested cells were inoculated onto regeneration medium viz. MS basal medium containing benzylaminopurine (3 mg l^−1^) and kinetin (1 mg l^−1^) further incubated at 26 ± 2 °C with 16/8 h photoperiod regimes (Fig. 5c). It was noticed that the proliferation of Q117 callus was much higher as compared to Co 86032 but the shoot regeneration was significantly superior in Co 86032 (41.4 ± 4.95 per 20 calli) (Fig. 5d) with respect to Q117 (10.6 ± 1.99 per 20 calli). The present results of plant regeneration from cell suspension through the embryogenic cells supports the observations of Vasil and Vasil (1982) and Ho and Vasil (1983) and also support the principle approach of regeneration in tissue and cell culture of the poaceae is passing through somatic embryogenesis. The roots were induced by incubating elongated shoots excised from plantlets developed from proliferating callus on MS basal medium supplemented with naphthalene acetic acid (Fig. 5e, f). It was observed that roots induced in Co 86032 and Q117 were comparatively equal in number and lengths as well as they were morphologically similar (Fig. 6a, b). The variation in proliferation, shoot and root regeneration response might be due to the influence of growth regulators and hormones on growing and developing callus (Salehi et al. 2014), genotypic and phenotypic characters of the mother plant (Benderradji et al. 2012). Due to the time necessity for whole plant regeneration, many authors (Ho and Vasil 1983; Ahloowalia and Maretzki 1983) did not mention clearly the process of plant regeneration. In other hand not many researchers have attempted to regenerate whole plants due to the difficulties associated with regeneration from suspension culture of sugarcane.

### Assessment of genetic stability using RAPD and ISSR

**Fig. 7:**
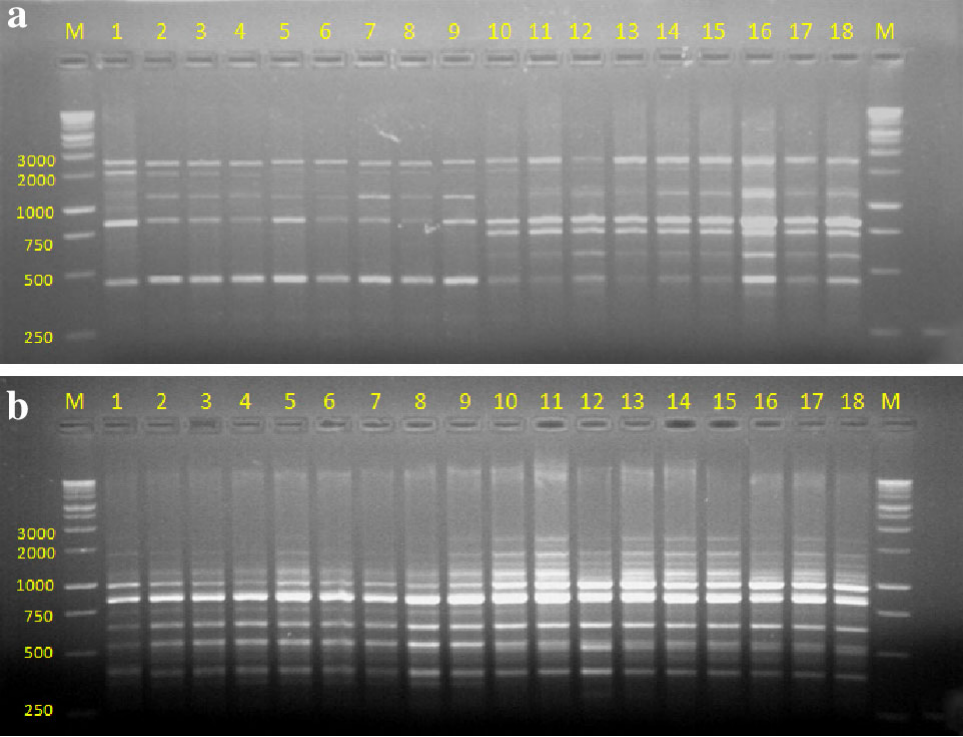
RAPD profile of cell suspension culture regenerated plants of sugarcane. **a** OPH-02. **b** OPH-06. *Lane M*: 1 kb marker,* 1*: parent of Co 86032,* 2*–*9*: Co 86032 plants from cell suspension culture,* 10*: parent of Q117 and* 11*–*18*: Q117 plants from cell suspension culture

**Fig. 8:**
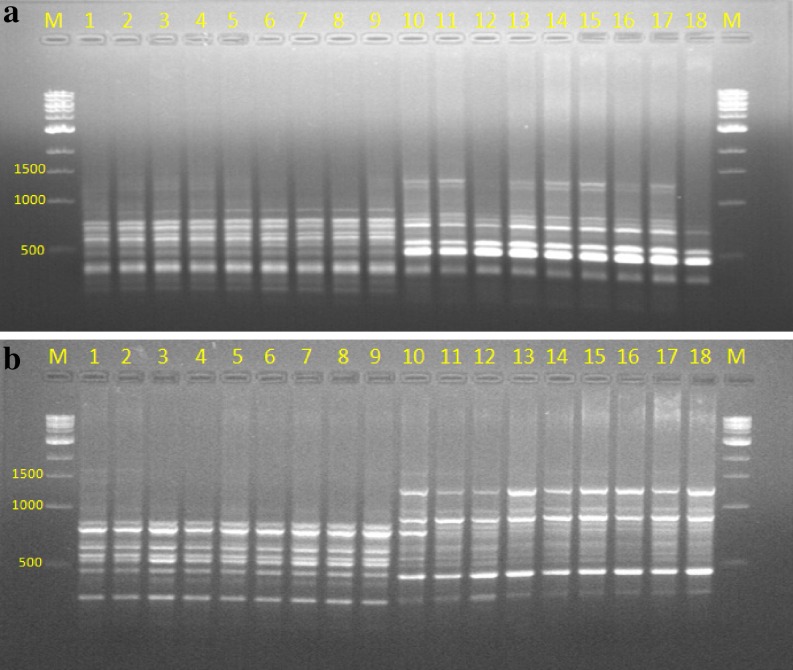
ISSR profile of cell suspension culture regenerated plants of sugarcane. **a** UBC 811. **b** UBC 857. *Lane M*: 1 kb marker,* 1*: parent of Co 86032,* 2*–*9*: Co 86032 plants from cell suspension culture,* 10*: parent of Q117 and* 11*–*18*: Q117 plants from cell suspension culture

The quality of total genomic DNA extracted from the young leaves of field grown mother plant and in vitro raised plants was determined by agarose gel electrophoresis using λHind III as molecular marker and quantity on UV–Vis spectrophotometer by recording OD at 260 and 280 nm. Genetic fidelity analysis using genomic DNA of the parent and in vitro raised plants was carried out to confirm genetic stability using RAPD and ISSR markers. The fingerprinting profiles of the parent and in vitro regenerated plantlets through cell suspension culture (Co 86032 and Q117) using the RAPD and ISSR markers produced distinct and reproducible amplified products (Figs. 7, 8) and scoring data are summarized (Tables 2, 3). RAPD analysis resulted in a total of 42 and 46 reproducible bands of Co 86032 and Q117 in vitro grown plants respectively (Table 2). The number of bands produced per RAPD primer was ranging from 2 to 6 in Co 86032 and 2–8 in Q117. The percentage of monomorphic bands in Co 86032 and Q117 were 90.48 and 86.96%, which polymorphic bands in Co 86032 and Q117 were 9.52 and 13.04% respectively. The size of amplified DNA fragments produced by using selected ten primers ranged from 250 to 3000 bp for Co 86032 and Q117 (Table 2). It was also observed that only three primers (OPH-02, OPH-09 and OPH-18) and five primers (OPH-01, OPH-04, OPH-06, OPH-13 and OPH-18) were showing polymorphism in Co 86032 and Q117 respectively. The monomorphic bands in RAPD represent the presence of the same allele at a particular locus, while polymorphic bands indicate two or more alleles at a single locus. The uniformity in banding pattern confirms the genetic fidelity of in vitro raised sugarcane plantlets from cell suspension culture.

Ten ISSR primers were selected for analysis after a preliminary screening of 10 RAPD primers. All the ten ISSR markers yielded 62 and 64 scorable and reproducible bands in Co 86032 and Q117 respectively. Each primer produced a unique set of amplification products ranging in size from 50 to 2000 bp in both the variety and number of bands produced per ISSR primer was ranging from 4 to 9 in Co 86032 and 5–9 in Q117 (Table 3). The percentage of monomorphic bands in Co 86032 and Q117 were 100 and 92.18% and the rest of the 7.81% polymorphic in Q117. It was also observed that only four primers (UBC-811, UBC-835, UBC-849 and UBC-857) were showing polymorphism in Q117. The uniformity in banding pattern confirms the genetic fidelity of in vitro raised sugarcane plantlets from cell suspension culture. The possible reasons for polymorphism observed during RAPD and ISSR analysis that occurred in in vitro raised plantlets regenerated from mother plants using cell suspension culture due to source of explants, mode of in vitro regeneration, duration of cell and tissue multiplication, accumulating mutations during the process of indirect organogenesis, etc. (Rizvi et al. 2012; Kshirsagar et al. 2015).

Martin et al. (2004) and Lakshmanan et al. (2007) suggested that use of multiple markers amplifying various regions of the genome, allowing high probability for the successful identification of polymorphism. RAPD (arbitrary, dominant) and ISSR (semi-arbitrary, medium to highly reproducible, dominant and more stringent) usually based on the non-coding regions of DNA. These two markers are simple, cost effective and proved efficient in evaluating the genetic homogeneity, genetic diversity and evolutionary studies (Harish et al. 2014; Rathore et al. 2014). RAPD and ISSR has been successfully applied to determine genetic fidelity/stability in several micropropagated plant systems viz. sugarcane (Suprasanna et al. 2006; Devarumath et al. 2007; Lal et al. 2008), *Chlorophytum borivilianum* (Rizvi et al. 2012), *Terminalia beelerica* (Dangi et al. 2014), *Aloe barbandesis* (Sahoo and Rout 2014), *Swertia lawii* (Kshirsagar et al. 2015), *Dendrocalamus strictus* (Goyal et al. 2015), *Dendrocalamus hamiltonii* (Singh et al. 2013) etc. These markers have been successfully applied for the detection of polymorphisms that are either induced or incorporated during in vitro regeneration of several plant species (Asthana et al. 2011). Ahloowalia and Maretzki (1983), Ho and Vasil (1983), and Falco et al. (1996) were developed plants from sugarcane cell suspension culture, however not mentioned about variation in the regenerated plants.

Based on the above observations, the plant regeneration through cell suspension culture was developed for both the varieties of sugarcane. This protocol imparts successful technique that can be utilized for developing somaclones and genetic trans-formation for its further improvement. This study clearly indicates that the RAPD and ISSR marker technique can be utilized for detection of genetic variation in early stages. To the best of our knowledge this is the first report which clearly mentions that the plants regenerated from sugarcane cell suspension culture and detects the genetic variation by using molecular markers.

## Electronic supplementary material

Below is the link to the electronic supplementary material.

Supplementary material 1 (DOCX 451 kb)
