# Supplementary material for: Plant regeneration from cell suspension culture in Saccharum officinarum L. and ascertaining of genetic fidelity through RAPD and ISSR markers
Source: 3 Biotech. 2017 Apr 8;7(1):16. doi: 10.1007/s13205-016-0579-3 (PMC5385170; doi:10.1007/s13205-016-0579-3)
Supplement: Supplementary file 1 — Supplementary material 1 (DOCX 451 kb) [file 13205_2016_579_MOESM1_ESM.docx]

**Supporting Figures:**

**
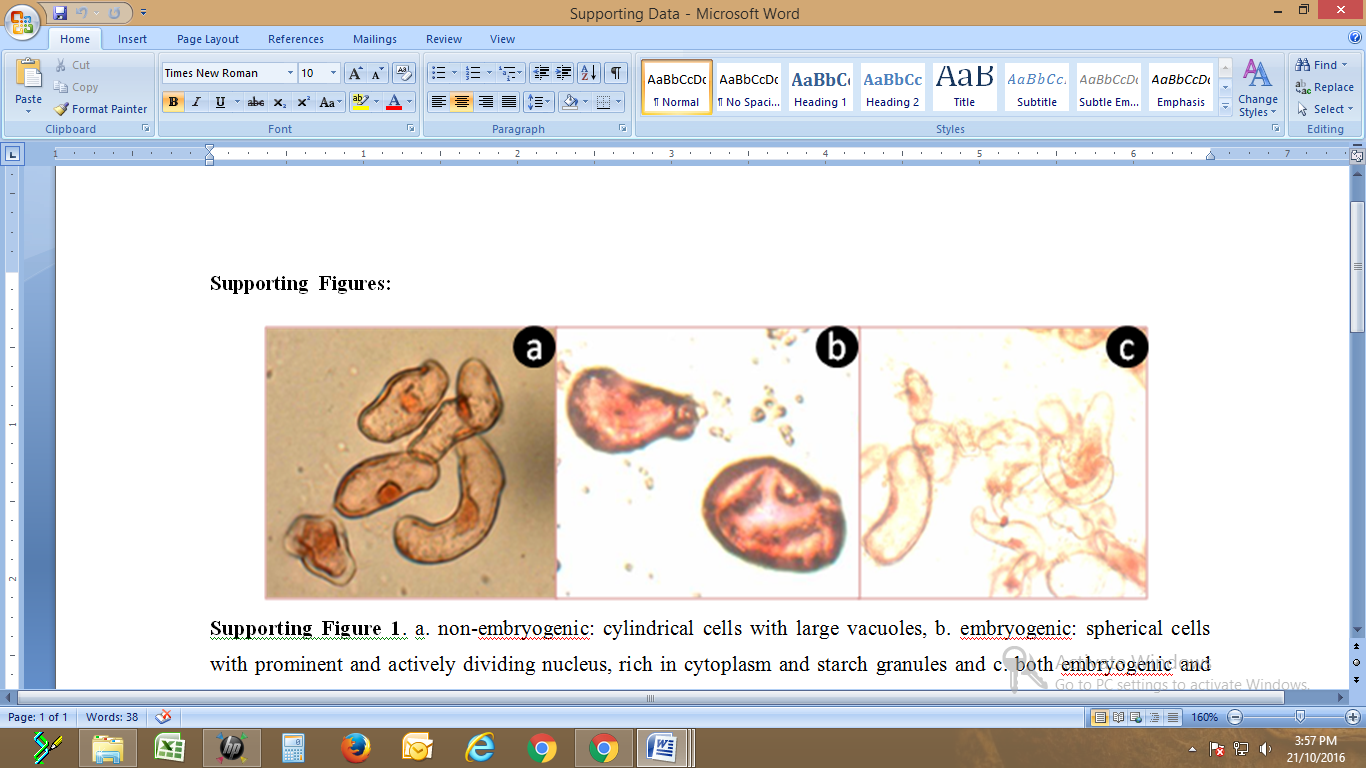
**

**Supporting Figure 1**. **a.** non-embryogenic: cylindrical cells with large vacuoles, and **b.** embryogenic: spherical cells with prominent and actively dividing nucleus, rich in cytoplasm and starch granules.
